# Antimalarial activity of *Syzygium guineense* during early and established *Plasmodium* infection in rodent models

**DOI:** 10.1186/s12906-016-1538-6

**Published:** 2017-01-05

**Authors:** Solomon Asmamaw Tadesse, Zewdu Birhanu Wubneh

**Affiliations:** Department of Pharmacology, School of Pharmacy, University of Gondar, Gondar, Ethiopia

**Keywords:** Malaria, *Plasmodium berghei*, *Syzygium guineense*, Antiplasmodial

## Abstract

**Background:**

In Ethiopia, the leaves of *Syzygium guineense* have been found useful for the prevention and cure of malaria, and demonstrated antiplasmodial activity in vitro. Nevertheless, no scientific study has been conducted to confirm its antimalarial activity in vivo. Therefore, the objective of the study was to evaluate the antimalarial effect of *Syzygium guineense* leaf extract in mice.

**Methods:**

Inoculation of the study mice was carried out by using the malaria parasite, *Plasmodium berghei*. The plant extract was prepared at 200, 400 and 600 mg/kg. Chloroquine and distilled water was administered to the positive and negative control groups respectively. Parameters like parasitaemia, survival time and body weight were determined following standard tests (4-day suppressive, Rane’s and repository tests).

**Results:**

*Syzygium guineense* crude leaf extract displayed considerable (*p* < 0.05) parasite suppression at doses of 600 and 400 mg/kg in a 4-day suppressive test with chemosuppressive value of 59.39 and 49.09% respectively. *S. guineense* crude leaf extract also showed dose-dependent schizontocidal activity in both the repository and curative tests. The extract also prevented body weight loss and prolonged survival date of mice significantly (*P* < 0.05) at the highest dose employed in the study. Qualitative chemical assay for *S. guineense* methanolic leaf extract revealed that the plant is endowed with different plant secondary metabolites exemplified by terpenoids, alkaloids, triterpenes, flavonoids, anthraquinones, tannins, glycosides, saponins and phenols.

**Conclusion:**

*Syzygium guineense* leaf extract possess antimalarial activity in mice. The test substance was found to be safe with no observable signs of toxicity in the study mice. The results of the present work confirmed the in vitro antiplasmodial finding and traditional claims in vivo in mice. Therefore, *Syzygium guineense* could be regarded as a potential source to develop safe, effective and affordable antimalarial agent.

## Background

Regardless of intensive efforts to get rid of malaria, the infection remains to be among the top health problems in Ethiopia [1]. Malaria, though widespread in tropical Africa, the healthcare coverage of the continent in general and the rural areas in particular is still inadequate. People dwelling in this area have time tested experiences regarding medicinal plants. As a result, people in the countryside, wholly or partially, use medicinal plants for prevention as well as cure of various disease conditions especially malaria. People give predilection to use herbal remedies due to the fact that traditional healers are easily accessible and generally respected by the community. This could be also a result of inadequate knowledge of the rural people regarding the modern healthcare institutions coupled with their belief in herbal remedies as being more affordable, safer and effective compared to the modern medicine [1–3].

In Ethiopia, malaria is the major cause of morbidity and mortality, with approximately 5–10 million cases per year. The disease causes not less than 60,000 deaths every year and accounts for about 17% of outpatient visits to health organizations, 8% of admissions and 29% of inpatient deaths [4, 5]. Furthermore, *P. falciparum* malaria parasite has recently developed resistance to almost all of the currently available medications used to treat the infection [6–8].

*Syzygium guineense* is a large evergreen flowering plant (family Myrtaceae). The plant is found widespread in the regions of Australia, Asia and Africa including Ethiopia [9].

Historically, antimalarial drug therapy is closely associated with traditionally used medicinal plants contributing like quinine; the first plant derived natural product (identified from Cinchona bark) is still employed in antimalarial therapy. Phytochemical compounds such as alkaloids, also found in the leaves of *S. guineense*, are commonly implicated for their antiplasmodial activity of many plants including Cinchona tree from which quinine, the first antimalarial drug, was isolated [10, 11]. In Ethiopia, leaf decoction of *S. guineense* is being used traditionally to treat malaria [12, 13].

Other secondary metabolites present in *S. guineense* implicated for their antimalarial activity include: Terpenoids [14–16], Phenols [17], Anthraquinones [18, 19], and Flavonoids [20]. *S. guineense* leaves are also recognized by their immunomodulatory [21], anti-oxidant, and anti-inflammatory properties [22].

Furthermore; the members of the genus *Syzygium* are well recognized for their antimicrobial, anti-inflammatory, antimalarial and larvicidal effect in mosquito vector [23]. The family Myrtaceae [24] and different species of *Syzygium* have shown promising antimalarial activity in vitro [25].

Despite the plant being endowed with the aforementioned secondary metabolites known for their antimalarial activity and in vitro claims, information regarding its in vivo antimalarial efficacy is extremely lacking. Therefore, this study was aimed in evaluating the traditional claim as well as reported in vitro efficacy of *S. guineense* in vivo in mice.

## Methods

### Collection and identification of the plant material

The fresh leaves of *S. guineense* (Wild) D.C. were collected near the town of Gondar in North Gondar administrative zone, Ethiopia, in February 2015. *S. guineense* as a potential medicinal plant was selected depending on the evidence provided by herbalists in Northwest Ethiopia. The antimalarial plant *S. guineense* was kindly authenticated by a taxonomist at the Ethiopian National Herbarium, Addis Ababa University and the Voucher specimen (voucher number ST02/2015) deposited for future reference.

### Experimental animals

Albino mice of either sex (25–30 g) were used for this study. All the study mice were sustained under standard environment (Temperature of 22 ± 3 °C, Relative Humidity of 50–70% and 12 h light/dark cycle), with food and water *ad libitum*. Mice were allowed to adapt themselves to the experimental environment for 7 days prior to the initiation of the actual experiment. All the experimental protocols were evaluated and approved by the institutional review board of the university.

### Parasite isolate

For the in vivo evaluation of *S. guineense* leaf extract, chloroquine sensitive *P. berghei* ANKA strain was used. The malaria parasite, *P. berghei,* was kindly provided by Ethiopian Health and Nutrition Research Institute (EHNRI) and was sustained by serial passage of blood from infected mice to non-infected ones on weekly basis.

### Extraction procedure

Fresh matured leaves of *S. guineense* (Wild) D.C. were collected, washed, air dried under shade and grinded using mortar and pestle to coarse powder. A total of 400 g powder was extracted by maceration for 72 h, filtered with wattman filter paper (150 mm size), and the residue extracted further two times by maceration with the same duration (72 h each). Finally, the combined filtrate was kept in an oven (40 °C) for concentration. Further drying process was carried out in a desiccator for removal of water. Finally, the dried extract was maintained at −4 °C till the beginning of the actual experiment.

### Phytochemical analysis of the crude extract

A preliminary phytochemical study of *S. guineense* leaf extract was performed using standard procedures [12, 13].

### Parasite inoculation

Blood were collected from the tail veins of previously *P. berghei* infected donor mouse having a parasite load of 30–37% to infect the mice. Subsequently, the blood was diluted in normal saline (0.9%) so that the final suspension contained 10^7^ infected red blood cells in each 0.2 ml of the preparation. Accordingly, mice were inoculated with 10^7^ *P. berghei* parasitized RBCs via the intraperitoneal route [26–28].

### Acute toxicity study

The safety of the extract when taken acutely was performed according to the OECD guideline 425. A fixed dose of 2000 mg/kg body weight of *S. guineense* crude leaf extract was administered to a single mouse via the oral route by gavage. Before the administration of the test substance, mice were prohibited from free access to food for 3 h. Similarly, food was with held for 1 h after extract administration. Following administration of the crude extract, mice were closely observed for an hour, occasionally for 4 h in a day (24 h) for a total of 14 days. Mice were closely monitored for the occurrence of any behavioral abnormalities indicated by decreased appetite, tremor, convulsion, increased saliva secretion, lacrimation, diarrhea, death as well as other manifestations of toxicity. In view of the fact that no death of the study mice at the test dose of 2000 mg/kg were observed, additionally 4 mice were sequentially dosed the same dose of the extract [29].

### Test on early malaria infection

The suppressive activity of *S. quineense* crude leaf extract was evaluated according to the Peter’s suppressive assay (4- day suppressive test). The Peter’s test is widely used in screening of plant extracts for their in vivo antiplasmodial activity via the use of two important parameters (blood parasitemia and mean survival time of mice) [26, 27, 30]. Mice were infected with 1 × 10^7^ *P.berghei* and kept in the same compartment before being assigned in 5 groups.

Following the infection, mice were arbitrarily distributed in to 3 extract treated & 2 control groups (each group containing 5 mice in a cage).

The crude leaf extract was administered at 200, 400 and 600 mg/kg and chloroquine 10 mg/kg body weight via the oral route once per day dose with gavage.

After three hours of parasite inoculation, mice were treated daily for 4 consecutive days. After treatment is completed (4^th^ day), thin blood smears were made by drawing blood from the tail of each mouse. Accordingly, each smear was fixed in methanol, and giemsa (10%) stained for subsequent determination of parasitaemia with a microscope (via counting 4 fields of about 100 RBCs in each field).

The difference between the mean value of the negative control group and those of the extract treated groups were determined to evaluate the activity of *S. quineense* extract in mice [28, 29, 31, 32].

$$ \mathrm{Activity} = 100\hbox{--} \left[\mathrm{Mean}\ \mathrm{parasitaemia}\ \mathrm{treated}/\mathrm{Mean}\ \mathrm{parasitaemia}\ \mathrm{in}\ \mathrm{negative}\ \mathrm{control}\right]\times 100 $$

$$ \mathrm{Percent}\ \mathrm{parasitaemia}=\left[\mathrm{No}.\ \mathrm{of}\ \mathrm{parasitized}\ \mathrm{R}\mathrm{B}\mathrm{C}/\mathrm{Total}\ \mathrm{no}.\ \mathrm{of}\ \mathrm{R}\mathrm{B}\mathrm{C}\ \mathrm{counted}\right]\times 100 $$

### Monitoring of body weight changes

Body weight of each mouse was recorded before infection (day 0) and on (day 4) using sensitive digital balance.

### Monitoring of mean survival time

Mortality of the study mice was closely observed daily and the number of days from the time of infection with *P. berghei* up to the incidence of death was recorded for every mouse in both the treatment and control groups during the follow up period. Mean Survival Time (MST) of all mice were determined using the formula [28]:

$$ \mathrm{M}\mathrm{S}\mathrm{T}=\mathrm{Sum}\ \mathrm{of}\ \mathrm{survival}\ \mathrm{time}\ \mathrm{of}\ \mathrm{a}\mathrm{ll}\ \mathrm{mice}\ \mathrm{in}\ \mathrm{a}\ \mathrm{group}\ \left(\mathrm{days}\right)/\mathrm{Total}\ \mathrm{number}\ \mathrm{of}\ \mathrm{mice}\ \mathrm{in}\ \mathrm{that}\ \mathrm{group} $$

### Evaluation of the extract on residual P. berghei infection

The residual infection protocol described by Peter [32] was employed to evaluate the prophylactic activity of the extract. Consequently, either sex of 25 mice were weighed and randomized into five groups of five mice each. Group I received 0.5 ml distilled water per kg body weight. Groups II, III and IV received 200, 400 & 600 mg/kg body weight of *S. guineense* extract orally respectively. The standard drug, (10 mg/kg/day chloroquine), was administered to Group V. Treatment continued for three successive days (from D_0_ - D_2_). On the 4^th^ day (D_3_), mice were infected with 1 × 10^7^ *P. berghei* infected red blood cells. Finally, after 72 h, parasitaemia was determined via blood smear.

### Evaluation on established infection (Rane’s test)

The curative potential of *S. guineense* was evaluated by employing the method described by Ryley and Peters (1970). On the 1^st^ day (day 0), mice were injected intraperitoneally with a standard inoculum of 1 × 10^7^ *P. berghei* infected RBCs. Seventy-two hours later, following confirmation of parasitaemia, the mice were randomly assigned into two control and three test groups, each group containing five mice per cage. The test groups were sequentially treated with the prepared leaf extract of *S. guineense* (200, 400 and 600 mg/kg/day). The standard drug (10 mg/kg/day chloroquine) and vehicle (0.5 ml distilled water) was administered to the positive and negative control groups respectively. Treatment continued for 5 consecutive days at a single dose per day. Geimsa stained (10%) thin blood film (prepared by drawing blood from the tail vein of each mouse) was examined microscopically to determine the percentage of parasitaemia.

### Statistical analysis

Results of the study were presented as mean ± SEM. Data was analyzed using SPSS version 20. Statistical significance was determined by One-way ANOVA coupled to Tukey’s HSD technique to compare result between doses and among treatment and control groups. For all the data obtained, the result was considered significant at 95% confidence level and *P*-value < 0.05.

## Results

### Phytochemical analysis

Preliminary phytochemical study of the extract of *S. guineense* (Wild) DC leaves revealed the presence/absence of plant secondary metabolites shown in Table 1.

**Table 1:** Phytochemical constituents of the extract

Phytochemical Constituents	Result
Polyphenols	+
Alkaloids	+
Saponins	+
Flavonoids	+
Tannins	+
Anthraquinones	+
Terpenoids	+
Triterpenes	+
Diterpenes	_
Cardiac glycosides	+
Steroids	_

Key: (+) = presence, (−) = absence

### Acute toxicity

*S. guineense* leaf extract didn’t cause death of the study mice at the limit dose of 2 g/kg. Similarly, both physical and behavioral observations of the study mice also did not point out any visible signs of toxicity. This indicates that the LD_50_ of the extract is above 2 g/kg.

### In vivo antimalarial activity tests

#### Test on early malaria infection (the 4-day suppressive test)

Leaf extract of *S. guineense* reduced parasitaemia level in the study mice dose-dependently at all tested doses. The crude extract, at the highest doses employed, (400 and 600 mg/kg per day) exhibited 49.09 and 59.39% chemosuppression respectively. Average parasite load at these doses were much lower (*p* < 0.05) than that observed in the untreated mice. The extract was also able to suppress parasitaemia (26.67%) at 200 mg/kg). The complete data is presented in Table 2.

**Table 2:** Effects of *S. guineense* crude leaf extract on early infection

Treatment	Group	Dose	% Parasitaemia	% chemosuppression
DW (negative control)	Group I	0.5 ml	33 ± 2.62	_
*S. guineense* leaf extract	Group II	200 mg/kg	24.20 ± 3.80^ns^	26.67
	Group III	400 mg/kg	16.80 ± 5.54^*^	49.09
	Group IV	600 mg/kg	13.40 ± 1.96^*^	59.39
Chloroquine (standard)	Group V	10 mg/kg	0.80 ± 0.37^*, **, ***^	97.57

*DW* distilled water

Values are indicated as means ± SEM, *n* = 5, ^ns^ = not significant, ^*^ = *p* < 0.05 compared to negative control, ^**^ = *p* < 0.05 compared to 200 mg/kg extract, ^***^ = *p* < 0.05 compared to 400 mg/kg extract

The comparison analysis indicated that the extract prevented weight loss significantly (*p* < 0.05) at all tested doses compared to distilled water treated control group. The increase in body weight was found to be highest at the largest dose used in the study (9.82%) (Table 3).

**Table 3:** Effects of *S. guineense* leaf extract against weight loss in early malaria infection

Treatment	Group	Dose	Wt. D-0	Wt. D-4	% change
DW (negative control)	Group I	0.5 ml	24.84 ± 1.55	23.96 ± 1.29	−3.54
*S.guineense* leaf extract	Group II	200 mg/kg	23.90 ± 1.13	24.88 ± 1.24	4.10^*^
	Group III	400 mg/kg	27.38 ± 0.66	27.76 ± 0.65	1.38^*^
	Group IV	600 mg/kg	26.46 ± 1.17	29.06 ± 0.74	9.82^*^
Chloroquine (standard)	Group V	10 mg/kg	25.39 ± 1.62	26.06 ± 1.43	2.63^*^

*DW* distilled water

Values are expressed as means ± SEM, *n* = 5, ^*^ = *p* < 0.05 compared to negative control

The extract prolonged mean survival period of the study mice (from 8 ± 1.73 to 10.8 ± 2.59 days). The corresponding vehicle treated mice survived only for 7 ± 0.70 days. Though much lower than the chloroquine-treated group (which lived for 28 days), the extract at 600 mg/kg was capable of significantly increasing (*P* < 0.05) survival date of mice (Table 4).

**Table 4:** Effect of *S. guineense* extract on survival time in the suppressive test

Treatment	Group	Dose	Mean Survival times (days)
DW (negative control)	Group I	0.5 ml	7 ± 0.70
*S. guineense* leaf extract	Group II	200 mg/kg	8 ± 1.73^ns^
	Group III	400 mg/kg	8.2 ± 1.30^ns^
	Group IV	600 mg/kg	10.8 ± 2.59^*^
Chloroquine (standard)	Group V	10 mg/kg	28 ± 0.00^*, **, ***, ****^

*DW* distilled water

Values are expressed as means ± SEM, *n* = 5, ns = not significant, ^*^ = *p* < 0.05 compared to negative control, ^**^ = *p* < 0.05 compared to 200 mg/kg, ^***^ = *p* < 0.05 compared to 400 mg/kg, ^****^ = *p* < 0.05 compared to 600 mg/kg of the extract

**Table 5:** Curative effect of *S.guineense* leaf extract against *P. berghei* infected mice

Treatment Dose (mg/kg)		Mean Parasitemia		Survival time (days)
		Pre –(D_3_)	Post –(D_7_) R_x_	
Control (−ve)	-	19 ± 0.62	43. ±0.76	7 ± 0.22
Extract	200	18 ± 0.63	17 ± 0.66	8 ± 0.72
	400	17 ± 0.73	13 ± 0.72^*^	13 ± 0.14^*^
	600	16 ± 0.81	6 ± 0.44^*^	15 ± 0.44^*^
Chloroquine	10	10 ± 0.83	2 ± 0.37^*^	28 ± 0.56^*^

Values are expressed as means ± SEM, *n* = 5, ^*^ = significant at *p* < 0.05 compared to the negative control

#### Test on prophylactic activity (repository test)

*S. guineense* extract exhibited a dose-dependent reduction in parasitaemia level in mice. At the highest dose used in the study (600 mg/kg body weight), the crude extract demonstrated a considerable (*p* < 0.05) antiplasmodial activity compared to the vehicle treated control group. Average chemosuppression at this dose was 48.57% which is lower than that exhibited by the standard drug (72.85%).

#### Test on established malaria infection (curative test)

*S.guineense* leaf exerted a considerable (*P* < 0.05) curative effect at 600 and 400 mg/kg. Mean parasite counts at these doses were 6 ± 0.44 and 13 ± 0.72 respectively. The extract at 200 mg/kg was also capable of decreasing parasite load to 17 ± 0.66 while, the mean parasite count in the untreated control group was 43 ± 0.76 (Table 5). Parasite load in extract treated mice was lower than that of the untreated mice indicating its pharmacological effect against established malaria infection. Nevertheless, this noticeable antiplasmodial effect of *S. guineense* was not comparable to the standard drug that reduced mean parasite density to 2 ± 0.37.

**Table 6:** Effect of crude leaf extract of *S. guineense* on residual malaria infection

Treatment	Group	Dose	%parasitaemia	%chemosuppression
DW (negative control)	Group I	0.5 ml	14.00 ± 4.04	_
*S. guineense* leaf extract	Group II	200 mg/kg	11.40 ± 1.16^ns^	18.57
	Group III	400 mg/kg	10.80 ± 1.62^ns^	22.85
	Group IV	600 mg/kg	7.20 ± 3.24^*^	48.57
Chloroquine (standard)	Group V	10 mg/kg	3.80 ± 1.32^*^	72.85

*DW* distilled water

Values are expressed as means ± SEM, *n* = 5, ns = Not significant compared to negative control, ^*^ = significant at *p* < 0.05 compared to the negative control

*S. guineense* leaf extract treated mice lived for 15 ± 0.44, 13 ± 0.14, and 8 ± 0.72 days at 600, 400 and 200 mg/kg/day dose respectively. On the contrary, mice receiving vehicle lived for 7 ± 0.22 days only. Chloroquine treated mice displayed the longest mean survival time (28 ± 0.56 days) which is significantly (*P* < 0.05) high compared to mice in both extract and distilled water treated groups.

## Discussion

In vivo screening of medicinal plants for their potential antimalarial activity is usually performed with a rodent malaria parasite, particularly *P. berghei,* which has been used extensively in the discovery and development of several conventional antimalarial drugs exemplified by halofantrine, chloroquine, mefloquine and more recently artemisinin derivatives [27].

Therefore, in the current study, antimalarial activity of *S.guineense* was evaluated using *P. berghei,* which produces disease similar to those of human *plasmodium* infections, for the prediction of treatment outcomes [31]. A standard antimalarial drug suppresses parasitemia significantly [33] which is in agreement with the effect of chloroquine in this study that achieved 97.57% suppression of the growth of *P. berghei* (the complete data is available in Table 2).

The 4-day suppressive assay is a standard test universally used for antimalarial screening, and the determination of percent inhibition of parasite growth is regarded as the most dependable parameter in antimalarial drug discovery [34]. In the 4-day suppressive test on *P. berghei* infected mice, *S. guineense* crude leaf extract exhibited dose-dependent activity at different doses employed in the study. The result is similar to other in vivo studies [33, 35–38]. A significant (*P* < 0.05) parasite suppression was observed at doses of 600 and 400 mg/kg compared to the negative control group. It is important to note that the crude extract at the lowest dose (200 mg/kg) also exhibited parasite suppression though not statistically different from the control group indicating that the extract is endowed with antiplasmodial activity and supports its traditional claim as antimalarial herbal remedy in Africa [23, 24].

The methanolic leaf extract of *S.guineense* also displayed antimalarial effect against residual infection of the parasite at all tested doses. The result of the study clearly indicated dose-dependent parasite suppression of *S.guineense* leaf extract even though not comparable with the chloroquine treated control group which suppressed 72.85% (Table 6). The low activity of the extract observed in the repository test when compared to the effect against early infection may be due to rapid clearance of the active agent by the liver.

The crude extract was also capable of increasing mean survival time (MST) of the study mice indicating that it suppressed the growth of *P. berghei*. Moreover; the extract was capable of reducing the overall pathological effects of this parasite in mice. It is also good to note that the survival time of the study mice receiving 600 mg/kg of the extract was significantly (*p* < 0.05) high as compared to those groups receiving 200 and 400 mg/kg as well as distilled water treated control mice. This may suggest a dose-dependent schizontocidal activity of the extract.

Body weight loss is one feature of rodent malaria infection. The extract not only prevented body weight loss in mice but also increased the weight of the study mice (Table 3). This indicates that other than direct parasiticidal effects, the plant may possess other pharmacologic benefits to the host: acting as analgesics, antipyretics, immune stimulators or the extract may contain appetite enhancing agent (s) [39].

Phytochemical screening of *S. guineense* leaf extract revealed the presence of alkaloids, terpenoids, anthraquinones, flavonoids, tannins, saponins, glycosides, triterpenes and phenols (Table 1). Alkaloids, also present in this plant, are generally known for their antiplasmodial activity of many plants including Cinchona bark from which quinine was isolated [21, 22]. Terpenoids have been implicated for their antiprotozoal and antimalarial activities in many pharmacological studies [21, 22]. *S. guineense* leaf also contain phenols known for their anti-oxidant and other diverse physiological properties: anti-carcinogenic, anti-inflammatory and anti-parasitic activities [10]. Anthraquinones, also found in this plant, is identified as active antimalarial compound [11, 14]. Flavonoids, also present in *S. guineense* leaf extract, are other potential antimalarial compounds believed to act by inhibiting the fatty acid biosynthesis (FAS II) of the malaria parasite [14]. *S. guineense* leaf possesses potential immunomodulatory [21], anti-oxidant [15, 40], anti-inflammatory and analgesic effects [22, 41]. *S. guineense* extract may also act via another unknown mechanism that may contribute for its antiplasmodial effect.

Therefore, these observed schizontocidal activities of *S. guineense* in mice could be attributed to the diverse classes of phytochemical compounds present in the leaf. On the other hand, the plant may be endowed with undiscovered (unknown) antimalarial compound (s) that could be used for the production of relatively safe, effective and affordable antimalarial agent.

## Conclusion

The present work has confirmed the efficacy of *S. guineense* supporting its traditional use against malaria. The results also indicated that the test substances have wide safety of margin making it potential source for the development of safer and cost-effective alternative drug in the treatment and cure of malaria.

## Abbreviations

ANOVA: Analysis of variance
DW: Distilled water
EHNRI: Ethiopian Health and Nutrition Research Institute
FAS: Fatty acid biosynthesis
LD_50_: Lethal dose 50 (Median lethal dose)
MST: Mean survival time
ns: Not significant
OECD: Organisation for Economic Co-operation and Development
RBC: Red blood cell
R_x_: Treatment
SEM: Standard error of the mean
